# Chronic Low-Level IFN-γ Expression Disrupts Mitochondrial Complex I Activity in Renal Macrophages: An Early Mechanistic Driver of Lupus Nephritis Pathogenesis

**DOI:** 10.3390/ijms26010063

**Published:** 2024-12-25

**Authors:** Heekyong R. Bae, Su-Kyung Shin, Ji-Yoon Lee, Yeo Jin Ko, Suntae Kim, Howard A. Young, Eun-Young Kwon

**Affiliations:** 1 Department of Food Science and Nutrition, Kyungpook National University, Daegu 41566, Republic of Korea; baehee@knu.ac.kr (H.R.B.);; 2 Center for Food and Nutritional Genomics, Kyungpook National University, Daegu 41566, Republic of Korea; 3 Omixplus, LLC., Gaithersburg, MA 20850, USA; 4 Cancer Innovation Laboratory, Center for Cancer Research, National Cancer Institute, Frederick, MA 21702, USA; 5 Center for Beautiful Aging, Kyungpook National University, Daegu 41566, Republic of Korea

**Keywords:** interferon gamma, mitochondrial complex I, macrophage dysfunction, lupus nephritis, autoimmune diseases

## Abstract

Mitochondrial dysfunction and macrophage dysregulation are well recognized as significant contributors to the pathogenesis of autoimmune diseases. However, the detailed mechanisms connecting these two factors remain poorly understood. This study hypothesizes that low but chronic interferon-gamma (IFN-γ) plays a critical role in these processes. To explore this, we utilized ARE-Del mice, a model characterized by sustained low-level IFN-γ expression and lupus nephritis (LN)-like symptoms. Age- and tissue-dependent gene expression analyses in ARE-Del mice revealed significant suppression of mitochondrial complex I components and activities, particularly in the kidneys. The genotype-dependent suppression of mitochondrial complex I indicates early disruption, which leads to macrophage dysfunction. Notably, remission restored gene expression of mitochondrial complex I and macrophage dysfunction in isolated renal macrophages from NZB/W lupus-prone mice. These findings suggest that chronic low-level IFN-γ disrupts mitochondrial complex I activity in macrophages, highlighting its role in the early pathogenesis of autoimmune diseases like lupus nephritis. This provides new insights into the molecular interactions underlying autoimmune pathogenesis and suggests potential targets for therapeutic intervention.

## 1. Introduction

Macrophage dysfunction is increasingly recognized as a key contributor to the pathogenesis of autoimmune diseases, including rheumatoid arthritis (RA) and systemic lupus erythematosus (SLE) [1,2]. Macrophages are polarized into two primary subsets: M1 and M2 [3,4]. M1 macrophages, activated by IFN-γ and microbial products, are pro-inflammatory and secrete cytokines such as tumor necrosis factor-alpha (TNF-α), interleukin-1 beta (IL-1β), interleukin-6 (IL-6), and interleukin-12 (IL-12), which contribute to pathogen clearance and promote inflammatory responses. M2 macrophages, on the other hand, are anti-inflammatory and involved in tissue repair, secreting cytokines like interleukin-10 (IL-10) and transforming growth factor-beta (TGF-β). M2 macrophages are also known to support the expansion and function of regulatory T cells (Tregs), further promoting immune tolerance and resolving inflammation [5]. An imbalance in the M1/M2 ratio, often skewed toward M1 dominance, has been observed in various autoimmune diseases [6]. However, recent evidence suggests that beyond this imbalance, abnormal macrophage function, including dysregulated signaling pathways and impaired interactions with Tregs, plays a more pivotal role in disease progression [7].

Mitochondria are central to regulating immune cell metabolism, and recent studies have identified mitochondrial dysfunction as a key driver of autoimmune diseases, including RA, SLE, and Sjögren’s syndrome [8,9,10]. Mitochondrial reactive oxygen species (ROS), generated during inflammatory responses, can lead to the oxidation of mitochondrial DNA (mtDNA) [11]. Oxidized mtDNA, when released into the cytosol, engages immune sensors such as cyclic GMP-AMP Synthase-Stimulator of Interferon Genes (cGAS-STING) and Toll-like receptor 7 (TLR7), triggering the production of type I interferons (IFNs) [12,13]. This overactivation of type I IFN signaling creates a positive feedback loop, amplifying inflammatory responses and promoting chronic inflammation, a hallmark of autoimmune pathology [14,15]. Furthermore, prolonged inflammation can interfere with the metabolic reprogramming of macrophages, preventing their transition from the pro-inflammatory M1 to the anti-inflammatory M2 phenotype [16]. This impaired metabolic switch in macrophages exacerbates their dysfunction, contributing to the sustained inflammatory environment seen in chronic autoimmune diseases. However, further research is required to fully establish these conclusions.

The mitochondrial respiratory complex consists of a series of protein complexes (I to IV) and ATP synthase (complex V) located in the inner mitochondrial membrane that are directly involved in oxidative phosphorylation (OXPHOS) [17]. Mitochondrial complex I, also known as NADH:ubiquinone oxidoreductase, plays a critical role in the transfer of electrons from NADH to ubiquinone, initiating the electron transport chain. The suppression of complex I activity can disrupt the normal flow of electrons, leading to a decrease in cellular energy production. The alteration in mitochondrial function, particularly in the electron transport chain (ETC), may contribute to obesity-related metabolic disturbances and insulin resistance [18]. Our recent paper reported that the inhibition of mitochondrial complex I plays a critical role in obesity-mediated chronic inflammation through the interaction between the liver and adipose tissue [19,20]. Given that mitochondrial dysfunction is well recognized in the pathogenesis of major autoimmune diseases such as type 1 diabetes (T1D), RA, multiple sclerosis (MS), and SLE [21,22], it is anticipated that this chronic inflammatory mechanism may also contribute to the development of autoimmune diseases.

IFN-γ is a key activator of M1 macrophages, driving their differentiation into the M1 phenotype and promoting inflammatory responses [23]. As a primary cytokine secreted by Th1 cells, IFN-γ is involved in the development of progression of several autoimmune diseases, including RA, SLE, and T1D [24,25]. However, the precise mechanisms underlying its contribution remain incompletely understood. Mitochondrial complex I activity is a fundamental mechanism triggering the IFN-γ response, with CRISPR-Cas9 genetic screening revealing its distinct role from other mitochondrial respiratory complexes [26]. In contrast, IFN-γ treatment in macrophages induces a metabolic shift, suppressing OXPHOS while enhancing anaerobic glycolysis to rapidly generate ATP [27]. This metabolic shift is crucial for Th1 cell activation and suppresses the differentiation of M2 macrophages [28]. Due to the intricate interplay of immune responses and the multifaceted nature of cellular signaling pathways, further research is critical to fully elucidate the underlying mechanisms and their implications for disease pathology.

ARE-Del mice, which exhibit systemic low but chronic expression of IFN-γ due to alterations in the AU-rich elements in the 3′-untranslated region (UTR) region, develop autoimmune diseases such as LN and primary biliary cirrhosis (PBC) [29,30]. Alterations in the AU-rich elements of the IFN-γ gene’s 3′-UTR lead to systemic, chronic low-level activation of IFN-γ that induces interactions with type I interferons, thereby exacerbating autoimmune responses [31]. Macrophage dysfunction, characterized by excessive oxidative stress and impaired fatty acid oxidation, is considered a central mechanism in the pathogenesis of these diseases [32]. A decrease in the NAD⁺ to NADH ratio in myocytes from ARE-Del mice suggests mitochondrial complex I inhibition, resulting in altered metabolism, reduced energy production, and increased oxidative stress, contributing to cardiomyopathy [33]. Therefore, utilizing this mouse model, further research is essential to elucidate the role of mitochondrial complex I in macrophage dysfunction and its contribution to autoimmune pathogenesis.

Here, our aim was to investigate whether chronic IFN-γ expression disrupts mitochondrial complex I, particularly in macrophages in the kidneys of ARE-Del mice, suggesting that this disruption is crucial for the pathogenesis of LN. We focused on how the disruption of mitochondrial complex I may inhibit a metabolic switch in macrophages, potentially leading to macrophage dysfunction. Furthermore, we examined whether these findings are consistent with other lupus-prone mouse models and clinical data, thereby validating the relevance of our results across different experimental and clinical contexts.

## 2. Results

### 2.1. Distinct Age- and Tissue-Dependent Hallmark Gene Set Expression in ARE-Del-/- Mice

**Figure 1:**
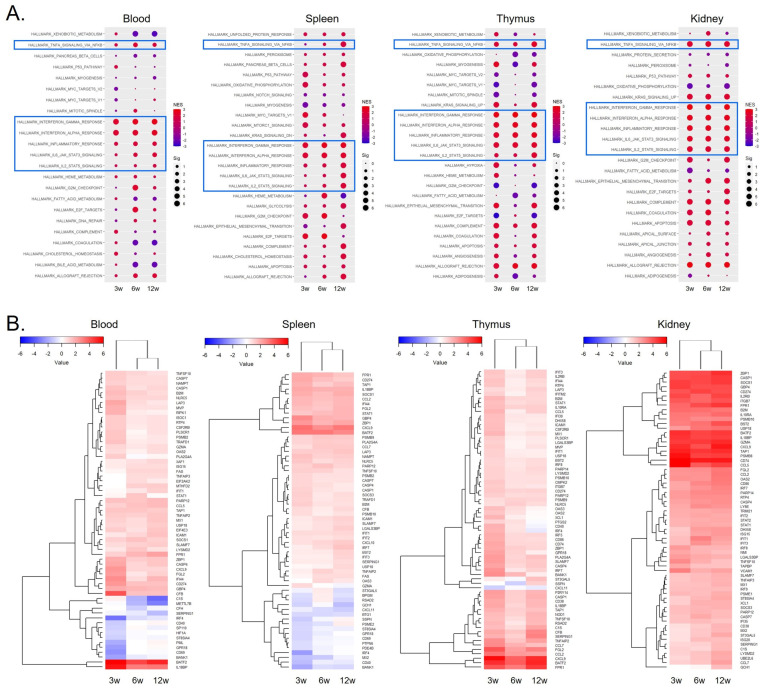
Time- and tissue-dependent hallmark gene set analysis using pre-ranked GSEA in ARE-Del-/- mice over time. (**A**) Hallmark gene set analysis at 3, 6, and 12 weeks in blood, spleen, thymus, and kidney of ARE-Del-/- mice. The dot size corresponds to the significance level (Sig), represented as −log10(FDR *q*-value), while the dot color indicates the NES. Blue squares highlight common inflammatory responses. (**B**) Heatmaps of IFN-γ response genes at 3, 6, and 12 weeks in blood, spleen, thymus, and kidney from ARE-Del-/- mice.

We performed pre-ranked GSEA to examine age-related variations in the hallmark gene sets across different tissues (Figure 1A). While distinct patterns were evident in various tissues, as highlighted by the blue rectangles, a common and statistically significant association with IFN-γ response and related inflammatory reactions was observed, similar to findings in obese subjects. Compared to the Enrichr results with DEGs, pre-ranked GSEA consistently produces more stable results across ages [32]. It is worth noting that distinctive changes at 6 weeks old were observed in all tissues, a commonality detected by both methodologies. This observed pattern of changes suggests a potential link between renal inflammation and the immune system via the blood stream. Tissue-dependent changes were also evident in the expression patterns of IFN-γ response genes in different tissues, as depicted in Figure 1B. When analyzing the trends within each tissue, a notably robust IFN-γ response was observed in the kidney. Overall expression profiles between the blood and spleen were similar, while the thymus and kidney had relatively higher expression of IFN-γ response genes.

### 2.2. Positive Correlation Between Suppressed Mitochondrial Complex I Activity and Macrophage Dysfunction in the Kidney of ARE-Del-/- Mice

**Figure 2:**
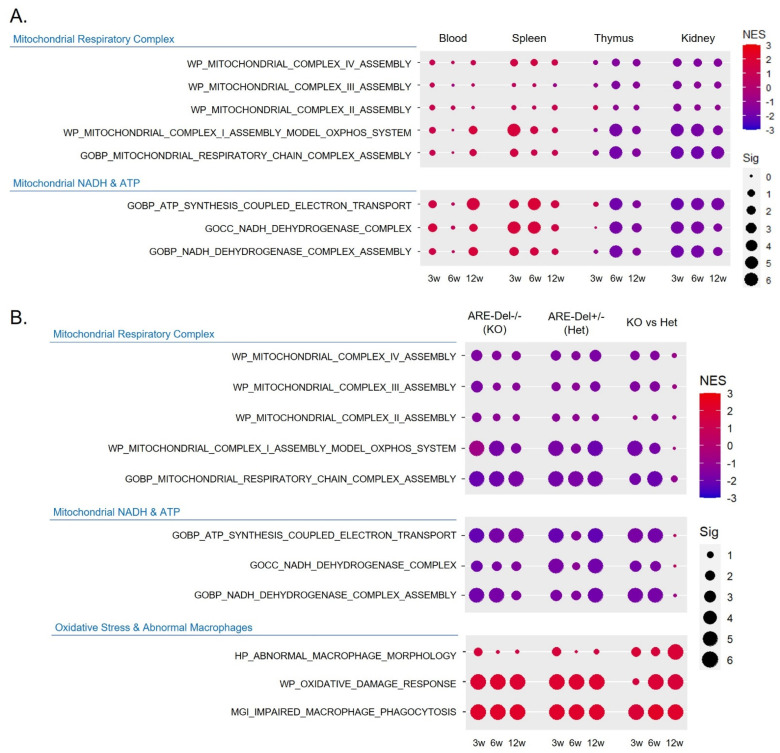
Dysregulated mitochondrial complex I activity in the kidney of ARE-Del-/- mice. (**A**) Tissue-dependent suppression of mitochondrial complex I activity in ARE-Del-/- mice. Dot plots demonstrating the enrichment of gene sets related to mitochondrial respiratory complexes and mitochondrial NADH and ATP responses in blood, spleen, thymus, and kidney of ARE-Del-/- mice at 3, 6, and 12 weeks of age. The dot size corresponds to the significance level (Sig), represented as −log10(FDR *q*-value), while the dot color indicates the NES. (**B**) Comparison of mitochondrial complex I activity in the kidneys between ARE-Del-/- vs. ARE-Del+/- mice at 3, 6, 12 weeks of age. Dot plots demonstrating the enrichment of gene sets related to mitochondrial respiratory complex, mitochondrial NADH and ATP, and oxidative stress and abnormal macrophages. The dot size corresponds to the significance level (Sig), represented as −log10(FDR *q*-value), while the dot color indicates the NES. Columns represent enrichment changes: KOs (ARE-Del-/- vs. control littermates), Hets (ARE-Del+/- vs. control littermates), and KOs vs. Hets (ARE-Del-/- vs. ARE-Del+/- mice).

Figure 2A illustrates changes in tissue-specific mitochondrial complex activity associated with age. The kidney exhibited the most significant reduction in mitochondrial complex I activity among complexes I, II, III, and IV across all ages, while the thymus showed a significant reduction after 6 weeks of age. In contrast, the blood and spleen demonstrated an opposite effect on mitochondrial complex I activity, although the significance was not very strong. These responses were confirmed through pathway analysis related to mitochondrial NADH and ATP responses, such as ATP synthesis coupled with electron transport and the NADH dehydrogenase complex.

Next, we conducted a comparative analysis of ARE-Del homozygotes (ARE-Del-/-) and heterozygotes (ARE-Del+/-) using pre-ranked GSEA. Figure 2B presents a time-dependent comparative analysis of ARE-Del homozygotes and heterozygotes. The first three columns represent changes in ARE-Del homozygotes (KOs) over time, followed by the next three columns representing ARE-Del heterozygotes (Hets), and the final three columns showing time-dependent changes in ARE-Del homozygotes versus heterozygotes (KOs vs. Hets). The suppression of complex I activity was most significantly impaired in both ARE-Del homozygotes and heterozygotes, with this response ongoing from the early stages. The initial suppression response was more pronounced in ARE-Del homozygotes than in heterozygotes. However, by 12 weeks, no significant difference was observed between the two groups, suggesting that both groups displayed maximal suppression.

### 2.3. Similarity of Hallmark Gene Sets Between Renal Inflammation in ARE-Del-/- Mice and Isolated Renal Macrophages from NZB/W Lupus-Prone Mice

**Figure 3:**
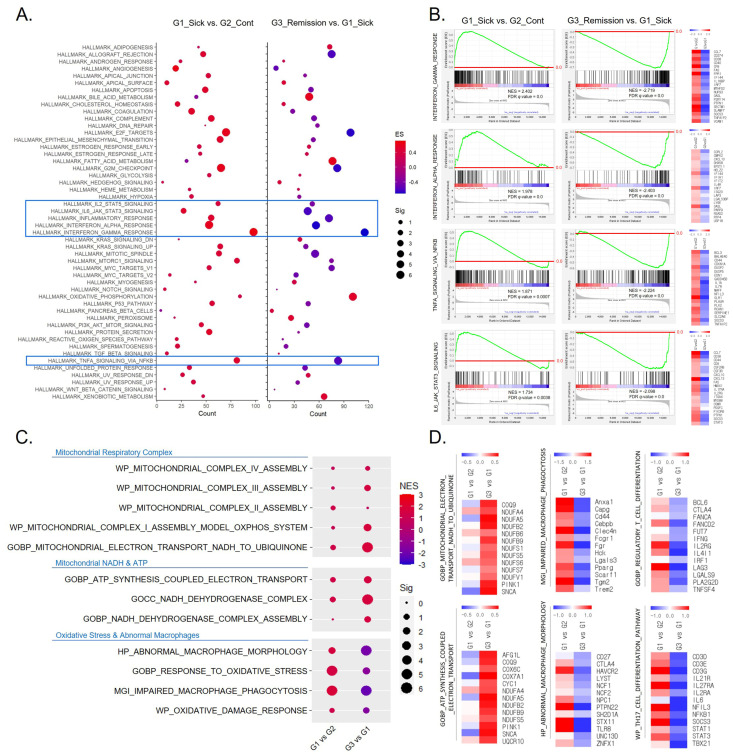
Recovery of inflammatory responses and complex I activity by remission in isolated renal macrophages from NZB/W lupus-prone mice. (**A**) Dot plots illustrating the changes in hallmark gene sets, comparing the groups of G1_Sick (the active phase of lupus nephritis) vs. G2_Control (during the onset of lupus) and G3_Remission (post-induction of remission) vs. G1_Sick. The y-axis represents fifty hallmark pathways. The count of genes significantly enriched in each pathway, determined by the GSEA algorithm, is shown on the x-axis. Dot size indicates significance through −log10(FDR *q*-value), while dot color reflects the ES, with red denoting a positive score and blue indicating a negative score. (**B**) Representative positively enriched plots resulting from GSEA of gene sets associated with IFN-γ, IFN-α, IL-6, and TNF-α responses, comparing the groups of G1 vs. G2 and G3 vs. G1. The baseline (0.0) of the enrichment score is indicated with a red-colored line. The heatmaps illustrate the expression levels of highly enriched genes within each gene set, allowing a comparison between two groups. (**C**) Dot plots demonstrating the enrichment of gene sets related to mitochondrial respiratory complex, and mitochondrial NADH and ATP, and oxidative stress and abnormal macrophages comparing the groups of G1 vs. G2 and G3 vs. G1. (**D**) Heatmaps displaying the expression levels of highly enriched genes within each gene set, allowing a comparison between two groups the groups of G1 vs. G2 and G3 vs. G1.

If the suppression of mitochondrial complex I activity serves as the initial trigger for the onset of mitochondrial dysfunction in the kidney, we assumed that similar results could be observed in isolated macrophages from the kidneys of other lupus-prone mouse models. To investigate this, as described in Section 4, we analyzed gene expression data from isolated F4/80(hi) macrophages from the kidneys of NZB/W lupus-prone mice during the onset of lupus (G2_Control), the active phase of lupus nephritis (G1_Sick), and post-induction of remission (G3_Remission). As shown in Figure 3A, the hallmark gene sets of G1 vs. G2 exhibited overall positively enriched patterns in the kidney, like what was observed in ARE-Del-/- mice, especially in terms of IFN-γ and related inflammatory responses. Conversely, during remission, triggered by a single dose of cyclophosphamide combined with six doses of CTLA4Ig/anti-CD154, isolated macrophages showed negatively enriched gene sets related to the IFN-γ response. As marked with a blue square in Figure 3A, the enriched patterns of gene sets related to IFN-γ, IFN-α, TNF-α, and IL-6 responses were similar to what was observed in obese individuals and ARE-Del-/- mice, as shown in Figure 3B. We have highlighted the top 20 significantly changed genes in each gene set, with red indicating an increase and blue indicating a decrease.

### 2.4. Recovery of Suppressed Complex I Activity by Remission in Isolated Renal Macrophages from NZB/W Lupus-Prone Mice

As the responses related to IFN-γ observed in isolated renal macrophages of NZB/W lupus-prone mice were similar to the changes in the macrophage-related gene sets in ARE-Del-/- mice mentioned earlier, we conducted a comparative analysis of complex I activity and its associated responses in isolated renal macrophages of NZB/W lupus-prone mice during the onset of disease, the active phase of lupus, and remission. It is important to note that the control group presented here does not represent a disease-free state but rather the onset of lupus. We anticipated that the suppression of complex I activity would likely occur from the early stages, which could explain the absence of significant differences between the active phase of lupus and the onset of the disease. As depicted in Figure 3C, there was no significant difference in overall mitochondrial complex activity between G1 and G2. However, in the comparison of G3 and G1, particular gene sets related to complex I activity were positively enriched. Upon analyzing various pathways associated with the suppression of complex I activity, our investigation revealed that the altered pathways in isolated renal macrophages during the active phase of lupus, as shown in Figure 3C, were oppositely regulated during remission. We also observed a significant reduction in the increased abnormal macrophage function during the active phase of lupus when transitioning into remission. Additionally, we selected an equivalent number of genes based on the highest degree of change from the selected gene list in Figure 3C, as presented in Figure 3D.

### 2.5. Suppressed Mitochondrial Complex I Activity and Macrophage Dysfunction in a Specific Renal Macrophage Cluster of LN Patients

Single-cell RNA sequencing of kidney specimens from LN patients revealed significant suppression of mitochondrial complex I activity within a distinct macrophage cluster. To investigate the correlation between IFN-γ signaling and the potential association with the development of autoimmune diseases, the expression levels of IFNG, IFNGR1, IFNGR2, HLA-DPA1, and HLA-DPB1 were analyzed across different cell clusters using the t-SNE plot (Supplemental Figure S1A). While IFNG was predominantly expressed in T cell clusters (T1, T2, T4, T5a), IFNGR2 was broadly distributed across B cells, endothelial cells, and macrophages, with IFNGR1 being distinctively concentrated in macrophages. Notably, IFNGR1 expression in macrophages mirrored the patterns of HLA-DPB1 and HLA-DPA1.

As shown in Supplemental Figure S1B, gene sets associated with mitochondrial complex I assembly were notably upregulated in epithelial cells (E0), activated B cells (B1), and dividing cells (D0). Conversely, these gene sets were downregulated in macrophages and T cells, with the M4 macrophage cluster showing the most pronounced suppression. The representative gene list related to ATP synthesis was also reduced in macrophages and T cells, particularly in the M4 cluster (Supplemental Figure S1C). Within the M4 macrophage cluster, there was a significant upregulation of genes involved in cholesterol metabolism and pathways associated with peroxisome proliferator-activated receptor gamma (PPARG) (Supplemental Figure S1D). Additionally, the M4 cluster displayed the highest expression levels of chemokine (C-C motif) ligand 2 (CCL2) and Fc gamma receptor I A (FCGR1A) (Supplemental Figure S1E), suggesting that M4 macrophages are primed for enhanced inflammatory activity.

## 3. Discussion

In this study on LN pathogenesis, we demonstrated that chronic low-level IFN-γ induced persistent suppression of gene expression related to mitochondrial complex I components and activities in a tissue-dependent manner within the kidneys. This early disruption was associated with alterations in the mitochondrial translational process, which may exacerbate mitochondrial dysfunction with metabolic imbalance. These findings were consistent with observations from isolated renal macrophages in lupus-prone mice and specific macrophage clusters identified through single-cell sequencing data from LN patients. Based on the above findings, we aim to discuss in the following section how IFN-γ can induce complex I inhibition and how this mechanism, in particular, can lead to dysregulation of macrophage function.

During inflammation, the metabolic switch from OXPHOS to glycolysis helps macrophages rapidly produce energy and metabolic intermediates necessary to support inflammatory responses and function effectively in an inflammatory environment [28]. While chronic IFN-γ may initially require mitochondrial complex I activity, as inflammatory responses escalate, its suppression can drive the shift from OXPHOS to glycolysis. Unlike type II IFNs, type I IFNs are known to depend on OXPHOS and fatty acid oxidation (FAO) for energy supply, particularly in plasmacytoid dendritic cells (pDCs) and non-hematopoietic cells [34]. In this context, the increase in type I IFNs induced by IFN-γ requires opposing metabolic pathways, but persistent inhibition of OXPHOS and FAO in macrophages can ultimately result in abnormal macrophage function. Considering the knockout of the type I IFN receptor (Ifnar1) or TLR7, which is particularly linked to the female-prevalent pathogenesis of PBC and LN in ARE-Del mice [30,31], the increase in type I IFNs induced by IFN-γ may be associated with macrophage dysfunction, likely due to the mitochondrial metabolic imbalances required for proper functioning.

This dynamic is also observed in the metabolic needs for the transition from M1 to M2 macrophages, further contributing to macrophage dysfunction. M2 macrophages primarily utilize OXPHOS and FAO as their main energy sources [35]. In contrast, M1 macrophages can induce rapid energy production, albeit less efficiently, by shifting from OXPHOS to glycolysis. In ARE-Del mice, there was a strong suppression of OXPHOS. Similarly, isolated renal macrophages from NZB/W lupus-prone mice also show marked suppression of OXPHOS. Interestingly, within the CM4 cluster, classified as M2-like macrophages from single-cell sequencing data of LN patients, there was a noticeable reduction in the expression of genes related to mitochondrial complex I components and activities. Additionally, our findings suggest that the opposing regulatory mechanisms between IFN-γ and PPAR-gamma (PPARγ) in macrophages could be linked to failures in controlling cholesterol transport and anti-inflammatory responses connected to the metabolic mechanisms discussed above. Overall, our findings suggest that the inhibition of the metabolic switch to anti-inflammatory responses, such as M2-type activation, may contribute to macrophage dysfunction in the pathogenesis of lupus nephritis.

Aside from metabolic switch imbalances, the underlying mechanisms of macrophage dysfunction in ARE-Del mice encompass several other aspects. Our prior research demonstrated that rapamycin treatment impairs autophagosome formation in isolated splenic macrophages from ARE-Del-/- mice [32]. This impairment is attributed to the inhibition of mTOR lysosome localization, which prevents mTOR activation by rapamycin, thereby disrupting the autophagosome formation process [36]. This is supported by evidence showing that mTOR lysosome localization is inhibited during macrophage priming induced by IFN-γ [37]. Moreover, impaired autophagosome formation can reduce DNA repair processes [38,39], leading to increased cellular apoptosis or senescence due to excessive oxidative stress induced by inflammation. Our data also reveal an upregulation of gene sets associated with cell death processes, including apoptosis and pyroptosis, as well as cellular senescence. Consequently, our findings suggest that defective autophagosome formation in ARE-Del mice may contribute to macrophage dysfunction and potentially to the pathogenesis of autoimmune diseases.

Autophagy dysregulation and metabolic switch imbalance are interconnected processes that can contribute to the development and progression of various diseases, including cancer and neurodegenerative diseases [40,41]. Autophagy serves a critical role in maintaining cellular homeostasis by degrading and recycling damaged organelles and proteins. When this process is disrupted, the accumulation of dysfunctional components, such as mitochondria, induces cellular stress and alters normal metabolic functions. This impairment interferes with the cell’s ability to efficiently manage energy demands, resulting in a shift toward an imbalanced metabolic state, often characterized by a reliance on glycolysis even under oxygen-rich conditions (akin to the Warburg effect observed in cancer cells) [42]. Such a metabolic switch imbalance may exacerbate the inflammatory environment characteristic of autoimmune conditions, as the altered energy metabolism fuels immune cell activation and perpetuates a cycle of inflammation and cellular dysfunction. Given evidence indicating that autophagy dysfunction can be involved in autoimmune disease pathogenesis [43], this bidirectional relationship between autophagy dysregulation and metabolic imbalance may play a pivotal role in sustaining and amplifying autoimmune pathology.

Next, regarding how the suppression of mitochondrial complex I leads to mitochondrial dysfunction in ARE-Del mice, when mitochondrial complex I activity is suppressed, ATP generation is reported to decline, and ROS levels are observed to rise, potentially leading to oxidative stress and damage to mitochondrial components, which drive the activation of the NLRP3 inflammasome [44,45,46]. This pathway is demonstrated in the kidneys of ARE-Del-/- mice, where chronic IFN-γ-induced suppression of mitochondrial complex I is associated with increased NLRP3 inflammasome activity and cell death, such as pyroptosis. Additionally, since cGAS-STING and TLR7 are known to induce type I interferon production, these pathways may also contribute to NLRP3 inflammasome formation and further amplify interferon signaling in ARE-Del mice. Mitochondrial stress or damage can lead to the leakage of oxidized mtDNA into the cytoplasm, where it activates the cGAS-STING pathway, promoting type I IFN production and strong inflammatory responses [47]. Similarly, TLR7 activation leads to the production of type I IFNs and other pro-inflammatory cytokines like TNF-α and IL-6 [48]. Therefore, chronic IFN-γ promotes the M1 macrophage transition, where suppression of mitochondrial complex I increases oxidative stress, leading to mtDNA leakage and type I IFN production, contributing to lupus development.

Additionally, one key finding from our study is that gene sets associated with mitochondrial complex I and mitochondrial translation are co-regulated. Given that some ETC components are directly regulated by mitochondrial translation [49], the suppression of mitochondrial translation could further inhibit the production of ETC components, including complex I, thereby exacerbating mitochondrial dysfunction. Since TLR7 primarily recognizes single-stranded RNA (ssRNA) as its ligand [50], the suppression of mitochondrial translation might generate fragmented ssRNA that could potentially trigger TLR7 signaling, driving LN pathogenesis. This raises the possibility that oxidized ssRNA fragments could activate innate immune pathways in a manner similar to that of oxidized mtDNA.

In conclusion, our study provides significant insights into the role of mitochondrial dysfunction in the pathogenesis of LN, highlighting how chronic IFN-γ-induced suppression of mitochondrial complex I activity contributes to metabolic imbalances, oxidative stress, and impaired macrophage function. The co-regulation between mitochondrial complex I and mitochondrial translation, coupled with the production of type I interferons and activation of inflammatory pathways such as the NLRP3 inflammasome and TLR7 signaling, underscores the intricate relationship between mitochondrial dysfunction and autoimmune responses. These findings suggest that targeting mitochondrial complex I activity and restoring mitochondrial function may represent a potential therapeutic strategy to mitigate macrophage dysfunction and, ultimately, the progression of LN and other autoimmune diseases. Further research into the interplay between metabolic reprogramming, autophagy dysregulation, and immune signaling will be essential in advancing our understanding of autoimmune pathogenesis and identifying novel intervention points.

## 4. Materials and Methods

### 4.1. Gene Expression of ARE-Del-/- Mice

ARE-Del-/- mice feature a 162 nt AU-rich element (ARE) region deletion in the IFN-γ gene’s 3′UTR, resulting in the exhibition of low but chronic expression of IFN-γ in the serum. Specifically, we have noted phenotypical similarities of LN and PBC autoimmune diseases in the kidney and liver, respectively [29,30]. As previously reported, RNA was isolated from spleen, thymus, kidney, and blood samples to analyze tissue- and age-dependent gene expression profiles influenced by genotypes (ARE-Del homozygotes and heterozygotes) [32]. The experiments, employing a minimum of three replicates, utilized samples obtained from both homozygous and heterozygous ARE-Del mice at 3, 6, and 12 weeks of age. These samples were compared with those from control littermates and were processed for hybridization using a custom Agilent array named Agilent-026855 (Agilent Technologies, Santa Clara, CA, USA). This array comprised 39,488 unique reporters and was executed according to the manufacturer’s protocol. The analysis utilized log2-transformed intensities that were normalized at the array level to maintain a consistent intensity spectrum. To assess the significance of gene expression differences among groups, the analysis employed ANOVA (Analysis of Variance) in Partek Genomics Suite 6.6 (v6.14.006). The original data used here were deposited in NCBI’s Gene Expression Omnibus (GEO) database under accession number GSE248465.

### 4.2. Renal Macrophage Data Acquisition

To validate that suppression of complex I activity is crucial for dysregulating macrophages in the kidneys of ARE-Del-/- mice, we performed a comparative analysis of gene expression profiles of the GSE27045 data set. As described by Bethunaickan et al., renal macrophages at different stages of nephritis were isolated using flow cytometry: isolated F4/80(hi) cells in the early stage of nephritis (control), during nephritis (sick), and after the induction of remission (remission) in the kidneys derived from nephritic NZB/W mice [51]. Samples were obtained from mice aged 8 to 16 weeks (n = 6) during the early stage, 2 to 6 weeks after proteinuria but before terminal renal failure (n = 7), and at remission induction, 3 to 4 weeks after achieving complete remission following a single administration of cyclophosphamide combined with six doses of CTLA4Ig and anti-CD154 (n = 4). The RNA extracted from the isolated cells was used for hybridization on the Affymetrix GeneChip^®^ Mouse Genome 430 2.0 array (Affymetrix, Santa Clara, CA, USA). To perform the differential expression analysis, we utilized GEO2R, using the t-test to evaluate significance between the means of two groups.

### 4.3. Pre-Ranked GSEA

Our previous research demonstrated the effective application of pre-ranked Gene Set Enrichment Analysis (GSEA) in investigating the mechanisms of obesity [19,20]. In pre-ranked analysis, gene expression profiles are ranked based on fold changes, and the GSEA algorithm is employed to assess gene set enrichment, utilizing two key statistical metrics: Normalized Enrichment Scores (NESs) and Enrichment Scores (ESs). Significance within the pre-ranked GSEA is commonly assessed through multiple criteria, including a nominal *p*-adjusted value, the False Discovery Rate (FDR), and the Family-Wise Error Rate (FWER), typically set at a threshold of less than 0.05. The FWER serves as a stringent measure to control the likelihood of incurring one or more false positives when conducting numerous hypothesis tests. In our analysis, we chose to base our assessment on the FDR, and in cases where a numerical value of ‘0’ was obtained, we added a minimal value of 1 × 10^−6^ before converting the result into a logarithmic representation. In our previous papers, we primarily relied on classical analysis for our results. However, in the case of ARE-Del-/- mice, where gene expression tends to be more robust, we employed weighted analysis to assess the significance of these changes. Weighted analysis assigns importance to changes at the individual gene level, potentially indicating higher significance, but it often exhibits lower NESs than classical analysis, emphasizing the need for cautious interpretation.

### 4.4. Acquisition of scRNA-Seq Data from Kidney Specimens of Patients with LN

We examined whether the findings from lupus mouse models correspond to observations in human patients by analyzing single-cell RNA sequencing (scRNA-seq) data from the kidney specimens of LN patients, utilizing interactive browsers available at https://immunogenomics.io/ampsle (accessed on 23 September 2024) and https://portals.broadinstitute.org/single_cell/study/amp-phase-1 (accessed on 23 September 2024) [52]. As summarized from the data collection and processing methods presented by Arazi, A. et al., kidney samples were collected from 24 LN patients and 10 control samples from living donor biopsies. Single-cell libraries were prepared using a modified CEL-Seq2 protocol, and sequencing was performed on the Illumina HiSeq 2500 (Illumina, San Diego, CA, USA), with reads aligned to the hg19 human genome using Spliced Transcripts Alignment to a Reference (STAR).

### 4.5. Data Visualization with R

We visualized the data primarily utilizing R, version R-4.3.1, and RStudio (version 2022.12.0 + 353) as our primary software tools. These sophisticated platforms, in conjunction with the versatility of packages such as ggplot2 (version 3.5.1) and heatmap2, (from the gplots package, version 3.1.3) empowered us to create a wide spectrum of informative and aesthetically pleasing graphical representations.

## Data Availability

The authors declare that all relevant data supporting the findings of this study are available in the paper and its supplementary information files or can be obtained from the corresponding authors upon request.

## Supplementary Materials

The following supporting information can be downloaded at: https://www.mdpi.com/article/10.3390/ijms26010063/s1.
