# Supplementary material for: Chronic Low-Level IFN-γ Expression Disrupts Mitochondrial Complex I Activity in Renal Macrophages: An Early Mechanistic Driver of Lupus Nephritis Pathogenesis
Source: Int J Mol Sci. 2024 Dec 25;26(1):63. doi: 10.3390/ijms26010063 (PMC11720139; doi:10.3390/ijms26010063)
Supplement: Supplementary file 1 [file ijms-26-00063-s001.zip › ijms-3334053-supplementary.pdf]

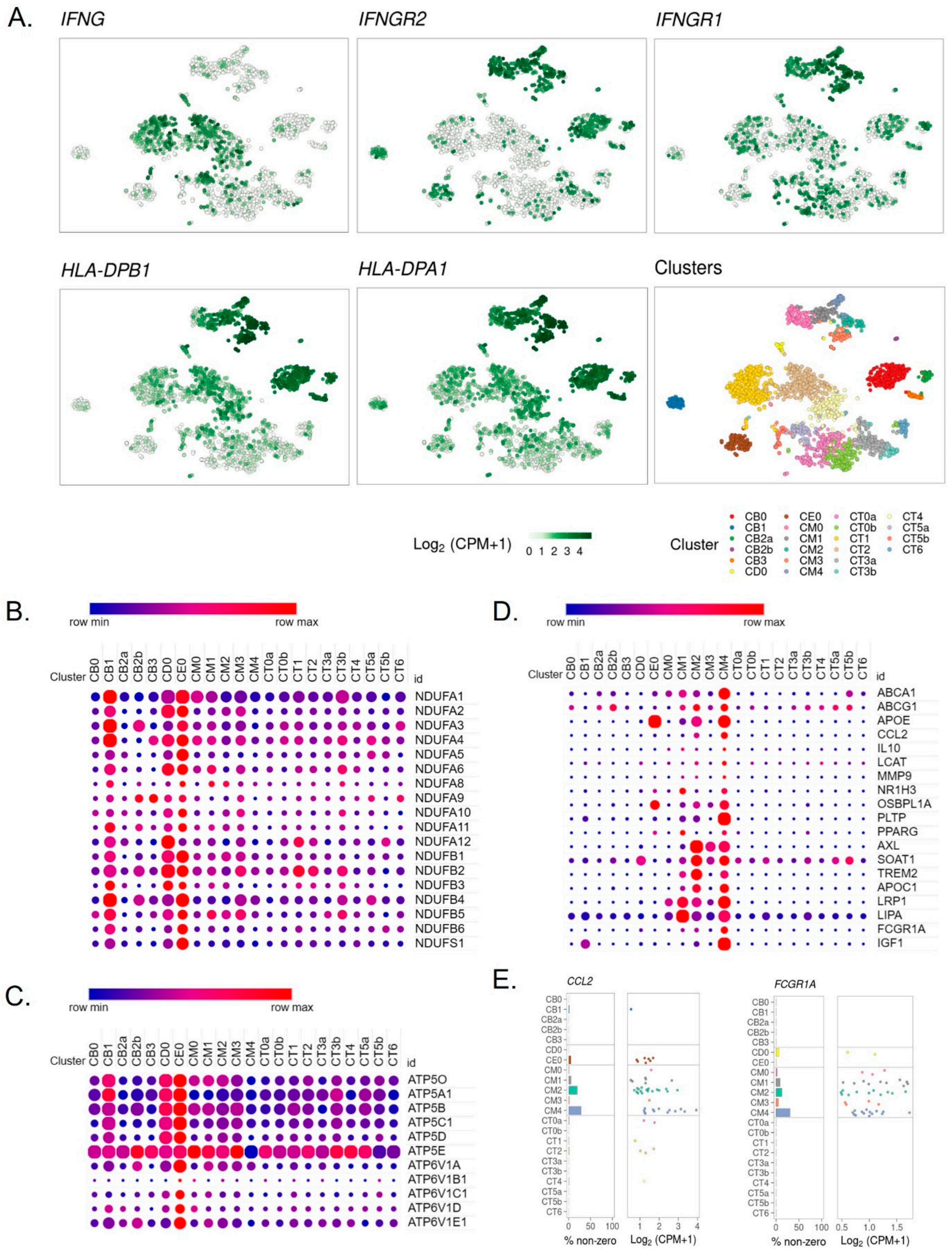


**Supplemental Figure 1. Single-cell RNA sequencing analysis of kidney specimens from LN patients.** (A) The t-SNE plot illustrates the distribution of IFNG, IFNGR1, IFNGR2, HLA-DPA1, and HLA-DPB1 gene expression across cell clusters, highlighting the unique expression patterns in

macrophages. Gene expression is indicated by green color, with darker shades representing higher expression (log2(CPM+1)). (B-C) Dot plots showing the expression of gene sets related to mitochondrial complex I assembly (B) and ATP synthesis coupled with electron transport (C) in each cluster. Scaling bar is relative to the intensity of gene expression across all cells within each annotation group. As the color shifts towards red, it indicates increased expression. (D) A dot plot showing the expression of gene sets related to PPARG-associated cholesterol metabolism pathways in each cluster. (E) Scatter plots showing the expression of CCL2 and FCGR1A in each cluster.

Cluster annotations [52]: CM0: CD16+ macrophage, inflammatory; CM1: CD16+ macrophage, phagocytic; CM2: Tissue-resident macrophage; CM3: cDCs; CM4: CD16+ macrophage, M2-like; CT0a: Effector memory CD4+ T cells; CT0b: Central memory CD4+ T cells; CT1: CD56_dim CD16+ NK cells; CT2: CTLs; CT3a: Tregs; CT3b: TFH-like cells; CT4: GZMK+ CD8+ T cells; CT5a: Resident memory CD8+ T cells; CT5b: CD56_bright CD16- NK cells; CT6: ISG-high CD4+ T cells; CB0: Activated B cells; CB1: Plasma cells/Plasmablasts; CB2a: Naive B cells; CB2b: pDCs; CB3: ISG-high B cells; CD0: Dividing cells; CE0: Epithelial cells
